# Remarks on Climate, and on Consumption

**Published:** 1817-05

**Authors:** Isaac Buxton


*For the London Medical and Physical Journal.*

### *Remarks on Climate, and on Consumption;* by Isaac Buxton, M. D.

SOME of your readers may recollect, that, several months ago, a controversy was carried on, in your Journal, between Dr. Sutton and myself, respecting the influence of climate on consumptive diseases. This was commenced by Dr. S. and afterwards discontinued by his desire. He has again commenced it, by endeavouring, in your Number for February, to overthrow positions, which, to say the least, I had rendered highly probable. Had he given the army-statements simply as he received them from Sir James M'Grigor, I should have taken no notice of the communication: but, when he accompanies those statements with remarks, which evidently are intended to diminish the force of the evidence I formerly brought forward, I should appear to to acquiesce in his conclusions were I to remain silent. As it is now nearly two years since this controversy was begun, it will be proper to remind your readers that the positions I advanced were,—1, that asthma and consumption are very rare in hot climates; 2, that asthma is rare, but consumption not unfrequent, in mild climates; 3, that they are both very prevalent in this country, the climate of which is cold and variable; 4, that in this country they are more frequent in winter than in summer; 5, that they have often been cured or relieved by the assistance of a high temperature (i. e. of about 65°) preserved in chambers during winter.

Of asthma, the term in common life generally employed to designate chronic catarrh or bronchitis chronica, Dr. Sutton has said nothing throughout the whole of what he has written; an indirect proof that he could not attempt to contradict my positions respecting this disorder.

Neither has he endeavoured to overthrow my assertion that rather more than one death in five in England is attributable to consumption.

In his late communication in your Journal for February, he says "that Malta has been mentioned as a climate in which consumption appears rarely. I, however, did not assent to this, from the circumstance of the disease having been observed to be common in Sicily."

Both Malta and Sicily are situated in the mild climate in which I have said that "consumption is not unfrequent." Dr. Sutton intends to convey the idea that consumption is more frequent in them than I allow: of course, that this disorder is nearly or quite as prevalent in them as in England. The opinion entertained by Dr. S. respecting Malta is no further entitled to respect than as it is supported by evidence, the doctor never having himself been in that island. Dr. Domeier, a German physician, who resided in Malta three years, wrote a small volume respecting that island, in which work he asserted that consumption was by no means so common there as in England. This Dr. Sutton has contradicted, and asserted that consumption is frequent there; not on the evidence of any person who had been in that island, but because, in Dr. S.'s opinion, Dr. Irvine affirmed consumption to be "common in Sicily." If Dr. Irvine had actually said this respecting Malta, the mind must still remain in doubt, as there would only be one evidence opposed to another. How much less can an inference be drawn respecting Malta, when Dr. Irvine's supposed assertion relates to another island! It is true, the two islands are near each other; but the circumstances in which they are placed are different. Malta is flat and dry, and its temperature very

 equable:

equable: Dr. Domeier asserts that he never saw snow there; in fact, that he had not known the thermometer to be lower than 51°. Sicily is mountainous, not unfrequently moist, the temperature irregular, and the falling of snow by no means an uncommon occurrence. Mr. Yeatman, in your Number for Nov. 1815, page 358, says, that "the climate of Sicily is subject to as frequent variations of temperature as that of England. I have often experienced the four seasons in Sicily in twenty-four hours." P. 359, he observes, that he "kept up a good fire from the middle of October to the latter end of December;" and yet "we found ourselves so chilly, even in the parlour, that we were often obliged to huddle on our great coats." To argue then that, because consumption is "common in Sicily," it must be frequent in Malta, is rather a strange mode of proof.

But, unfortunately for Dr. Sutton's opinion, consumption is not nearly so common in Sicily as in England. He has endeavoured to prove, by the authority of Dr. Irvine, the frequency of phthisis in that island. But I have shewn that, if the general tenor of Dr. I.'s book on the Diseases of Sicily is attended to, my opinions on this point, and not Dr. Sutton's, are evidently supported; and that Dr. S.'s supposed proof depends entirely on his separating particular passages; and, by detaching them from their situation, giving them a force, which, in their connexion, they do not possess. We have also other testimony besides that of Dr. Irvine. At page 359 of your Number for November 1815, Mr. Yeatman says, "As far as my observations went, that lamentable disease (consumption) was by no means so common in that island (Sicily) as in England. The symptoms of phthisis are oftener mitigated, and their deadly course oftener arrested, than in our own country." And, lower down, in the same page, he singles out one particular period when phthisis attacked some of the soldiers. When Sicily was threatened by Murat, they "were exposed to the vicissitudes of heat, and subjected to much corporeal fatigue and mental anxiety. Under these circumstances it was that several cases of phthisis occurred amongst our men towards the November and December of the same year; and, of these, a few recovered that would, in my apprehension, have died in England." Dr. Farrel, who was in Sicily many years, and of whose name Dr. Sutton, I believe, is not ignorant, says, in a letter to Dr. Forbes, of Argyll-street, that, "if one death in five be caused by consumption in England, I should suppose that not more than one in ten is produced by the same cause in Sicily." Mr. Pearse, of the Commercial Road, who was in Sicily a few years, has written to me, that he he thinks the deaths by consumption in that island are comparatively about half as numerous as in this country. The opinions of Mr. Yeatman, together with the ratio of Dr. Farrel and Mr. Pearse, of one death in ten by consumption in Sicily, while the deaths in England, by the same disease, are one in five, exactly correspond with my position "that consumption is not unfrequent in mild climates," but "very prevalent in this country;" and consequently it has not been "observed to be common in Sicily, and, *a fortiori,* not to be common in Malta.

Dr. Sutton observes, that he had been furnished by Sir James M'Grigor with the returns of deaths among the troops stationed in Malta and in Jamaica. By these it appears, that one death in five among the soldiers in Malta was occasioned by consumption; and, in Jamaica, one death in ten. This, he says, "is positive evidence on the subject, and shews the mortality by consumption in Malta to be great," and "cannot warrant the conclusion that," in Jamaica, "it is an unfrequent disease." These returns, at first sight, appear to favour Dr. Sutton's opinions; but, when more attentively considered, will, I think, be allowed to strengthen the positions I have advanced.

The great body of the soldiery is exactly of the consumptive age. All infantile diseases, and all diseases of old age, are unknown in the army. Hence some of the great sources of mortality do not appear in the returns from the troops; and, consequently, the diseases of the middle period of life exist in a very increased ratio. These opinions are supported by actual observation. The Philadelphia Bills of Mortality are drawn up with more accuracy than any others which I have seen. They are made out chiefly from the returns of medical men. The total number of deaths in the bill for the year 1815 is stated to have been 2049; of whom 347 died by consumption, which is nearly in the ratio of one in six. The number of persons who died between twenty and thirty years of age was 254, of whom 103 died by consumption, which is about one in two and a half. The number of deaths, by all diseases, occurring between ten and forty years of age, was 650; of whom the number of deaths by consumption was 237, equal to about one in three. The principal part of the army on service in foreign countries might be assumed to consist of persons between eighteen and thirty years of age; but, that I may include drummers and the older soldiers, I will allow them to consist of persons between ten and forty years old. In the Philadelphian Bill of Mortality we see that the proportion of deaths by consumption, including all ages, is one in six; the proportion between ten and forty years of age one in three, which is double that of the total. Hence, if the returns of the army give one in five as the ratio of deaths by consumption in Malta, and one in ten as the ratio in Jamaica, this would make the ratio of deaths among persons of all ages one in ten in the former, and one in twenty in the latter, island. Such a proportion exactly accords with the opinions which Dr. Farrel and Mr. Pearse have given respecting Sicily, and very nearly with the result of the tables of mortality in Jamaica furnished by Lempriere; which tables formed a principal foundation, on which I built my assertion, that consumption was rare in hot climates. Probably we should not be very far from the truth, were we to assume the return from the army as our guide, and to say, in a general way, that one in ten dies by consumption in mild climates, and one in twenty in hot climates.

Before closing this paper, I beg to return my thanks to you, Sir, for pointing out to me a source of information respecting Madeira, which I had overlooked. The observations and opinions of such a man as Dr. Adams must always be acceptable to me; but cannot fail of being highly grateful, when they strengthen opinions which, from other sources, I had formed. I likewise request Mr. Yeatman to accept my grateful acknowledgments for the account of Sicily given by him in your Journal, not merely because his observations confirm my assertions, but because he has contributed to settle points under discussion, which only could be settled by the testimony of those who had been eye-witnesses of what occurred in the countries in question. There cannot be a doubt that there are, in this island, great numbers of gentlemen well qualified, by their opportunities and talents, to verify or to contradict opinions which have been maintained respecting different countries. It appears to me, that, were they to volunteer their services, by giving the result of their observations on the diseases of different countries, their labours would be highly acceptable to your readers, and useful to the profession.

*New Broad-street;*

*March* 31, 1817.

